# Production of Plant Secondary Metabolites: Examples, Tips and Suggestions for Biotechnologists

**DOI:** 10.3390/genes9060309

**Published:** 2018-06-20

**Authors:** Gea Guerriero, Roberto Berni, J. Armando Muñoz-Sanchez, Fabio Apone, Eslam M. Abdel-Salam, Ahmad A. Qahtan, Abdulrahman A. Alatar, Claudio Cantini, Giampiero Cai, Jean-Francois Hausman, Khawar Sohail Siddiqui, S. M. Teresa Hernández-Sotomayor, Mohammad Faisal

**Affiliations:** 1 Research and Innovation Department, Luxembourg Institute of Science and Technology, 5 avenue des Hauts-Fourneaux, L-4362 Esch/Alzette, Luxembourg; jean-francois.hausman@list.lu; 2 Department of Life Sciences, University of Siena, via P.A. Mattioli 4, 53100 Siena, Italy; berni10@student.unisi.it (R.B.); giampiero.cai@unisi.it (G.C.); 3 Trees and timber institute-National research council of Italy (CNR-IVALSA), via Aurelia 49, 58022 Follonica (GR), Italy; cantini@ivalsa.cnr.it; 4 Unidad de Bioquímica y Biología Molecular de Plantas, Centro de Investigación Científica de Yucatán, Calle 43 # 130 X 32 y 34, Col. Chuburná de Hidalgo, Mérida, Yucatán 97205, Mexico; arms@cicy.mx (J.A.M.-S.); ths@cicy.mx (S.M.T.H.-S.); 5 Arterra Biosciences srl/Vitalab srl, via B. Brin 69, 80142 Naples, Italy; fapone@arterrabio.it; 6 Department of Botany & Microbiology, College of Science, King Saud University, P.O. Box 2455, Riyadh 11451, Saudi Arabia; eabdelsalam@ksu.edu.sa (E.M.A.-S.); ahmadaqq@gmail.com (A.A.Q.); aalatar@ksu.edu.sa (A.A.A.); 7 Life Sciences Department, King Fahd University of Petroleum and Minerals (KFUPM), 31261 Dhahran, Saudi Arabia; ksiddiqui@kfupm.edu.sa

**Keywords:** secondary metabolites, *Artemisia*, artemisinin, *Urtica dioica* L., lignans, *Coffea arabica* L., caffeine, bioactivity, heterologous hosts, uridine diphosphate glycosyltransferases, cell wall

## Abstract

Plants are sessile organisms and, in order to defend themselves against exogenous (a)biotic constraints, they synthesize an array of secondary metabolites which have important physiological and ecological effects. Plant secondary metabolites can be classified into four major classes: terpenoids, phenolic compounds, alkaloids and sulphur-containing compounds. These phytochemicals can be antimicrobial, act as attractants/repellents, or as deterrents against herbivores. The synthesis of such a rich variety of phytochemicals is also observed in undifferentiated plant cells under laboratory conditions and can be further induced with elicitors or by feeding precursors. In this review, we discuss the recent literature on the production of representatives of three plant secondary metabolite classes: artemisinin (a sesquiterpene), lignans (phenolic compounds) and caffeine (an alkaloid). Their respective production in well-known plants, i.e., *Artemisia*, *Coffea arabica* L., as well as neglected species, like the fibre-producing plant *Urtica dioica* L., will be surveyed. The production of artemisinin and caffeine in heterologous hosts will also be discussed. Additionally, metabolic engineering strategies to increase the bioactivity and stability of plant secondary metabolites will be surveyed, by focusing on glycosyltransferases (GTs). We end our review by proposing strategies to enhance the production of plant secondary metabolites in cell cultures by inducing cell wall modifications with chemicals/drugs, or with altered concentrations of the micronutrient boron and the quasi-essential element silicon.

## 1. Introduction

Plants are renewable resources providing raw material (like lignocellulosic biomass; [1]) and phytochemicals (notably secondary metabolites) for different industrial applications, namely in the textile, construction, pharmaceutical, nutraceutical and cosmetic sectors. Because of these features, plants are considered essential to favor the transition to a bio-economy that is less dependent on fossil resources.

Plants synthesize a huge variety of secondary metabolites, with complex chemical composition, which are produced in response to different forms of (a) biotic stresses, as well as to fulfil important physiological tasks, like attracting pollinators, establishing symbiosis, providing structural components to lignified cell walls of vascular tissues [2]. Importantly, many of the secondary metabolites produced by plants are used by pharmaceutical industries (since these *bioactive compounds* trigger a pharmacological or toxicological effect in humans and animals), in cosmetics, nutrition, for the manufacture of drugs, dyes, fragrances, flavors, dietary supplements. Hence, both the scientific and industrial interest around plant secondary metabolites is enormous.

In this review, we emphasize the huge variety of molecules of plant secondary metabolism by describing examples of terpenoids, phenolic compounds and alkaloids that, although specific, can give an overview of the many possible fields of application of these molecules. We draw the attention to known medicinal plants, such as representatives of *Artemisia*, as well as more neglected species, like stinging nettle (*Urtica dioica* L.). We discuss the production of secondary metabolites in response to exogenous stresses, by choosing the specific case of caffeine production in cell cultures of *Coffea arabica* exposed to Al. We survey the production of artemisinin and caffeine in heterologous hosts, as well as discuss some biotechnological strategies used to increase the bioactivity of plant secondary metabolites, by taking as example the use of glycosyltransferases (GTs, EC: 2.4.1.x). Finally, we conclude our review by providing suggestions that can be applied in plant biotechnology to increase the production of specific secondary metabolites, namely induction of cell wall stress in plant cell cultures. Several reviews have been published on plant secondary metabolites, covering both their production and applications [3] and the characterization of the phytochemical families occurring in different species [4,5]. However, to the best of our knowledge, this is the first survey proposing potential avenues for the increased production of secondary metabolites via induction of cell wall modifications.

## 2. Production of the Sesquiterpene Artemisinin in *Artemisia* and Heterologous Hosts

*Artemisia* is one of the largest plant genera belonging to the Asteraceae family with more than 500 species [6]. This family contains several species ranging from woody shrubs to herbaceous perennials, characterized by high levels of chemical compounds in their essential oils. *Artemisia* species are characterized by extreme bitterness of all parts of the plant [7]. They are mostly perennials [8]; however, about 10–20 species are annuals or biennials [9]. Furthermore, the growth habit of *Artemisia* spp. range from herbs to subshrubs and shrubs [10]. *Artemisia* is distributed worldwide and often occurs as the dominant type in some plant communities including steppe, semi-desert and desert steppe [8]. In coastal plains or ranges, they are distributed mainly on uncultivated hillsides in lower diversity [11]. Many species of *Artemisia* have a high economic value as ornamentals, food, soil stabilizers in disturbed habitats, or a good feed for several animals [12]; some taxa are toxic or allergenic, while some others are invasive weeds which can adversely affect crop yield [13,14]. This genus has always been of great medicinal interest and is useful in traditional remedies for a treatment of a variety of diseases [15]. *Artemisia* species have antimalarial, antitumor, antioxidant, antiviral, antipyretic, antihemorrhagic, anticoagulant, antianginal, antihepatitis, antispasmodic, antiulcerogenic, antifungal, interferon-inducing activities [14,16], as well as anti-inflammatory [17], antibacterial [18], antiepileptic and anticonvulsant [19] properties.

Plant cell and tissue culture techniques are being used widely for in vitro manipulation and re-vegetation of a large number of species for commercial purposes, including many medicinal plants. In many cases, it provides an opportunity to maintain true-to-type plant species and the propagation system can produce a large number of plants from a single clone with enhanced artemisinin contents. In vitro manipulation of different *Artemisia* species such as *A. annua* [20,21,22,23], *A. sieberi* [24], *A. vulgaris* [25,26], *A. japonica* [27], *A. nilagirica* var. *nilagirica* [28], *A. absinthium* [29,30], *A. abrotanum* [31], *A. amygdalina* [32], *A. carvifolia* [33], *A. aucheri* [34], *A. scoparia* [35], *A. judaica* [36], *A. absinthium* [37], *A. chamaemelifolia* [38] and *A. pallens* [39,40] has been attempted for various purposes. Some investigations were tried to increase the number of glandular trichomes as the organ responsible for accumulation of artemisinin in *A*. *annua* plants. Transferring the β-glucosidase gene via *Agrobacterium* in *Artemisia* plants increased the density of glandular trichomes in flowers and leaves and consequently enhanced the artemisinin content by 2.5% in flowers and 1.4% in leaves [41]. The in vitro culture techniques were also used to produce artemisinin in cell suspension and hairy root cultures [42]. Manipulations of growth conditions including various sugar concentrations, chilling treatment and UV-B radiation stimulated the production of artemisinin in *A*. *annua* tissue cultures [43,44,45]. Moreover, treatment of various elicitors including methyl jasmonate, gibberellic acid, salicylic acid and chitosan increased the production of artemisinin in different tissue cultures [46]. Mass production of artemisinin in bioreactors is now achieved via *A*. *annua* hairy root cultures using various cultivation and elicitation methods [47]. After 25 days of cultivation, this method produces more than 6 g of *A*. *annua* dry weight with 0.32 mg g^−1^ artemisinin content. Attempts to introduce the genes involved in the biosynthesis of artemisinin in heterologous hosts were successful; however, they led to very low yield [48]. *Nicotiana* spp. are the only plants used as heterologous host for artemisinin production studies, due to their high biomass and rapid growth with low cost [42]. Farhi et al. [49] transferred a single vector containing five different genes from the mevalonate and artemisinin pathways to *N*. *tabacum* and successfully produced artemisinin with a concentration of 0.48–6.8 µg g^−1^ dry weight. Expression of amorpha-4,11-diene synthase, amorphadiene monooxygenase, aldehyde Δ (13) reductase and aldehyde dehydrogenase genes in *N*. *tabacum* produced 0.01 mg g^−1^ dry weight of artemisinic alcohol in leaves [50]. Malhotra et al. [51] transferred six genes from the mevalonate pathway to *N. tabacum* chloroplast and the artemisinin genes pathway to the nuclear genome and successfully produced transgenic plants able to produce about 0.8 mg g^−1^ dry weight of artemisinin. The moss *Physcomitrella patens* has been recently introduced as a new heterologous host for artemisinin production [52]. Ikram et al. [42] transferred all five genes involved in the biosynthesis of artemisinin in *P*. *patens* producing 0.21 mg g^−1^ dry weight of artemisinin after 3 days of culture. 

**Figure 1:**
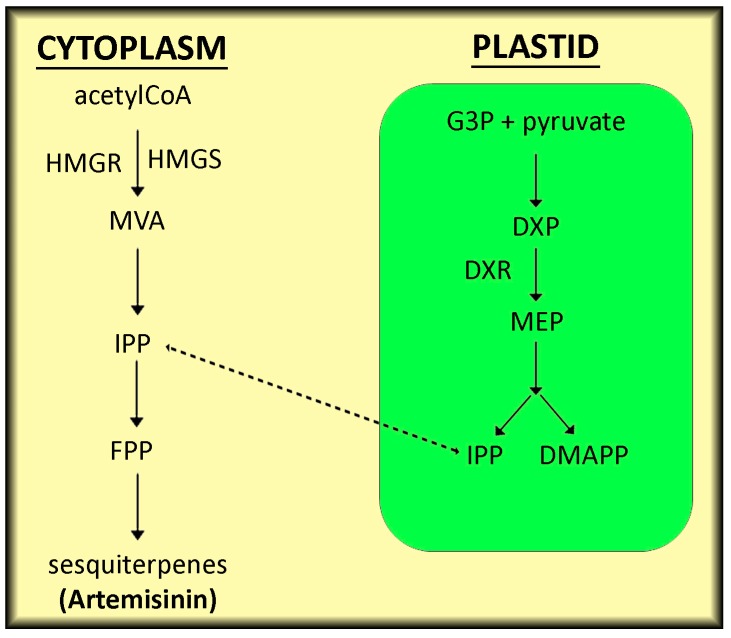
Diagram showing the biosynthesis of artemisinin via the mevalonate pathway in plant cells. Acetyl coenzyme A (acetylCoA), 3-hydroxy-3-methyl-glutaryl-coenzyme A reductase (HMGR), 3-hydroxy-3-methyl-glutaryl-coenzyme A synthase (HMGS), mevalonate (MVA), isopentenyl pyrophosphate (IPP), farnesyl diphosphate (FPP), glyceraldehyde 3-phosphate (G3P), 1-deoxy-d-xylulose-5-phosphate (DXP), 1-deoxy-d-xylulose-5-phosphate reductoisomerase (DXR), 2-*C*-methyl-d-erythritol 4-phosphate (MEP), dimethylallyl pyrophosphate (DMAPP).

Several studies investigated the chemical composition of different *Artemisia* species (Table 1). Nevertheless, the most important metabolite produced by *Artemisia* is the sesquiterpene lactone artemisinin, which is considered to be a high-efficient anti-malarial drug, besides its protective effects against cancers and viral diseases [53]. *A*. *annua* produces high amounts of artemisinin if compared to other *Artemisia* species. In this plant, artemisinin is mainly synthesized in the glandular secretory trichomes on leaves and flowers [54]. Putative transcription factors involved in the development of glandular secretory trichomes and the biosynthesis of artemisinin include ENHANCER OF GLABRA3 (Aa-EGL3) and TRANSPARENT TESTA GLABRA1 (Aa-TTG1) [55]. Furthermore, a trichome-specific fatty acyl-CoA reductase (Aa-TFAR1) gene that plays a role in cuticular wax biosynthesis, artemisinin regulator 1 (Aa-TAR1), which is member of AP2/ERF transcription factor superfamily and the AaMYB1 transcription factor, contribute significantly to the biosynthesis of artemisinin in the glandular secretory trichomes of *A*. *annua* plants [56,57]. Isopentenyl diphosphate (IPP) and dimethyl allyl diphosphte (DMAPP) derived from the cytosolic mevalonate (MVA) pathway are the precursors of artemisinin in plants. These precursors are derived from acetyl-CoA and the plastidic 2-*C*-methyl-d-erythritol 4-phosphate (MEP) pathway stemming from glyceraldehyde-3-phosphate and pyruvate [58]. IPP production depends upon MEP formation from 1-deoxy-d-xylulose-5-phosphate (DXP) in plastids which is catalyzed by 1-deoxy-d-xylulose-5-phosphate reductoisomerase (DXR) (Figure 1). The over-expression of DXR gene (AaDXR) increased the yield of artemisinin in *A*. *annua* [59,60]. Moreover, inhibition of DXR using fosmidomycin decreased the artemisinin content in *A*. *annua* plants by 25% [58]. IPP produced via the MVA pathway serves as a precursor for the biosynthesis of farnesyl diphosphate (FPP), which is required for the biosynthesis of sesquiterpenes, triterpenes and sterols [61]. In general, the biosynthesis of FPP via the MVA pathway is catalyzed by several enzymes including acetoacetyl-CoA thiolase (ATOT), 3-hydroxyl-3-methyglutaryl CoA synthase (HMGS), 3-hydroxyl-3-methyglutaryl CoA reductase (HMGR), mevalonate kinase (MK), mevalonate-5-phosphate kinase (MPK) and mevalonate pyrophosphate decarboxylase (MPD). Co-expression of genes encoding HMGR and FPP synthase (FPPS) increased the artemisinin content by more than 2 fold [61]. *A*. *annua* plants were supplied with labelled HMG-CoA, which enhanced the artemisinin content from 7.5 to 17.3 nmol (up to 130%) and it was proven that growth regulators, such as IAA and GA_3_, enhanced artemisinin biosynthesis and accumulation via an increase in HMGR activity [62]. Furthermore, cytochrome P_450_ enzymes play a critical role in the biosynthesis of artemisinin [61]. Co-expression of amorpha-4,11-diene 12-monooxygenase (CYP71AV1) with the genes involved in the biosynthesis of amorphadiene in *Saccharomyces cerevisiae* led to the presence of artemisinic acid in the culture [63]. Expression analysis of CYP71AV1 in *A*. *annua* plants revealed the over-expression in trichomes and flower buds, as compared to leaves and roots [64]. Indeed, CYP71AV1 accumulation in *A*. *annua* leaves is 8-fold higher if compared to the accumulation in roots and 4-fold higher if compared to the accumulation in stems [65]. Moreover, cloning and co-expression of cytochrome P_450_ reductase (CPR) with CYP71AV1 in yeast increased the concentration of artemisinic acid in the culture [66].

The characterization of genes and their encoded enzymes involved in the biosynthesis of artemisinin in *A*. *annua* plants paved the way to engineer the production of this valuable metabolite in cells of both plants and microorganisms. Several studies indicated that over-expression of terpenoid genes encoding HMGR, FPPS and DXR increased the production of artemisinin in *A*. *annua* [80,81,82,83,84]. Over-expression of CYP71AV1, CPR, amorpha-4,11-diene synthase (ADS), aldehyde dehydrogenase 1 (ALDH1) genes in *A*. *annua* transgenic plants led to 3.4-fold increase in the production of artemisinin [85]. Silencing of reactions that compete with the biosynthesis of artemisinin such as squalene and β-caryophyllene biosynthesis is considered to be another effective strategy to increase artemisinin production [42]. Chen et al. [86] found that silencing of β-caryophyllene synthase in *A*. *annua* plants increased the artemisinin production by 54.9% in transgenic plants, as compared to wild-type ones. Furthermore, Zhang et al. [87] suppressed the expression of squalene synthase in the sterol pathway, which led to an increase of 3.14-fold in the content of artemisinin if compared to non-transgenic *A*. *annua* plants. Another strategy for increasing artemisinin content in *Artemisia* plants is the regulation of its biosynthesis using *Agrobacterium rol A*, *B*, and *C* genes. These genes increase the contents of stress response metabolites in plant cells. Genetic transformation of different *Artemisia* species to express *rol* genes resulted in overexpression of artemisinin biosynthesis pathway and increased the artemisinin content in these plants [88,89,90]. Shen et al. [91] stated that the overexpression of several transcription factors regulating the biosynthesis of artemisinin, such as AaWRKY1, increased the artemisinin content in engineered plants with a 4.4-fold compared to control ones; overexpressing the jasmonate-responsive AaERF1 and AaERF2 transcription factors increased instead the expression of ADS, CYP71AV1, and artemisinic aldehyde delta-11(13) reductase (DBR2) genes and finally increased the accumulation and content of artemisinin in *A*. *annua* plants.

The production of artemisinin in plants has currently witnessed vast advances especially in the field of genetic and metabolic engineering. Microbial biosynthesis of artemisinic acid shows a significant potential for the industrial-scale production of artemisinin [42]; however, the relatively high cost required for producing artemisinin using this acid hinders the application of this process. The significant progress achieved in different techniques of plant genetic transformation, transcription factor engineering and cellular compartment targeting increased the potential of using such techniques with the aim to increase the production levels of artemisinin in different *Artemisia* species. The application and implementation of these techniques will enable the mass production of artemisinin in bioreactors ensuring the continuous production of such drug to meet the global demand.

## 3. Valorizing Stinging Nettle for the Production of Phenolic Compounds

Stinging nettle is a perennial plant found in temperate regions which produces phloem fibres rich in crystalline cellulose [92,93], as well as phytochemicals [94]. Despite its potential multi-purpose utilization, this plant has not received, so far, the attention it deserves: only a few studies are available on nettle, dealing with fibre yield [93], phytoremediation potential [95], carbohydrate analysis of micro-dissected tissues [96] and phenolic compound profiling [97]. We wish here to draw the attention on this neglected plant, with the goal of highlighting its enormous potential in the production of secondary metabolites. 

The leaves and stalks of nettle plants contain phenolic compounds, in particular the stalks are rich in flavonoids and anthocyanins, while the leaves contain hydroxycinnamic acid derivatives (chlorogenic acid and 2-*O*-caffeoylmalic acid) [97]. Isolated fibres contain hydroxycinnamic acid derivatives (probably dihydrosinapoyl alcohol) which come from lignin. The presence of lignin-derived phytochemicals in the fibres is interesting, as it provides a further way of valorizing nettle cultivation for the production of fibres and the extraction of phytochemicals for e.g., biomedical applications. It should be noted in this respect that nettle extracts were shown to possess antimicrobial properties. A study tested the antibacterial and antifungal activities of nettle crude extracts obtained from leaves and stems against 28 bacteria, 3 yeast and 7 fungal strains and showed that 47% of the extracts inhibited Gram-negative and > 60% had effects on Gram-positive bacteria, with ethyl acetate extracts showing the highest antimicrobial activity [98]. 

Aqueous extracts of nettle roots are used for the treatment of prostatic disease and ethanolic extracts showed effects on benign prostatic hyperplasia (BPH) [99]. In particular, these effects are due to the presence of a specific class of secondary metabolites, the lignans (phenylpropane dimers originating from the phenylpropanoid pathway [100]), which bind to the human sex hormone globulin and act as hormone balancer [101]. The production of lignans is usually achieved via plant cell suspension and hairy root cultures [102,103,104,105,106]. Their production in heterologous systems is not yet a viable option, since not all the players partaking in the pathway leading to specific lignin formation are known [107]. 

An increased lignan production can be achieved via precursor feeding: *Linum album* hairy root cultures fed with coniferaldehyde showed an increase in lariciresinol, pinoresinol and podophyllotoxin of ca. 15-, 9- and 1.5-fold, respectively [104]. Methyl jasmonate (MeJA) elicitation was used in *Isatis indigotica* hairy root cultures to increase the production of lignans (pinoresinol, lariciresinol, secoisolariciresinol, coniferin) and to identify the transcription factors involved in their production [105]. This approach led to the identification of AP2/ERFs transcription factors, as well as biosynthetic genes upregulated during the treatment. This approach is very interesting for the discovery of master regulators that could be eventually overexpressed for the metabolic engineering of cell suspension cultures, in a manner analogous to what described in *Forsythia* x *intermedia* [108]. Given the presence of lignans in the roots of stinging nettle, it would be interesting to establish hairy root cultures via transformation using *Agrobacterium rhizogenes* and to elicit the cultures with compounds such as MeJA or feed coniferaldehyde to boost the production of lignans. 

## 4. Production of Caffeine in Coffee Cell Cultures and Heterologous Hosts

Secondary metabolites are produced by plants in response to pathogen attacks, elicitors and have wide commercial and industrial applications [109]. 

A better understanding of the perception mechanisms triggered by external signals that can modify the levels of secondary metabolites can be valuable for the biotechnological implementation of more resistant plants to stress conditions [110], or to improve their production [111]. The synthesis of these chemical compounds using in vitro cultures provides an excellent area for in-depth investigation of biochemical and metabolic pathways, under highly controlled environmental conditions. Different environmental stress factors such as temperature, light and metals, that often increase the accumulation of secondary metabolites in plants, can be used in in vitro cell cultures [112,113].

Caffeine (1,3,7-*N*-trimethylxanthine) is a purine alkaloid that has been used widely by our modern society as a stimulant beverage in coffee, tea, and energy drinks. One of the many benefits of caffeine is that it increases alertness and prevents fatigue. Indeed, it is used as an ingredient to improve athletic performance in aerobic and anaerobic conditions. However, due to its inherent addictive characteristics, caffeine can be considered now one of the most consumed drugs all around the globe. 

More than 60 different types of plants produce caffeine as a natural pesticide, to protect themselves from insects. *Coffea arabica* L. (Arabica type coffee) is commercially grown on an important scale and it is recognized by the excellent commercial value provided by its beans [114,115]. In vitro *C. arabica* suspension cells have been developed to study different aspects that can regulate the production of caffeine [116] under several types of stress such as Al toxicity. In this model, Al inhibits cell growth, affecting the production of second messengers involved in the regulation of calcium homeostasis and protein kinase activities involved in in growth regulation [117,118]. Recently, the influence of Al on the production of caffeine was studied [117,118]. It was observed that when the cell cultures were kept in the dark, caffeine was not detected. However, when the cells were irradiated and supplemented with theobromine, these conditions allowed studying the effect of Al on caffeine metabolism. When the cells were treated with Al (500 µM), an increase of caffeine levels in the culture media, as well as into the cells, was detected. A significant increase in caffeine synthase activity was detected under Al-treatment in *C. arabica* cells, as well as the expression of the corresponding gene [119]. This suggests that the regulatory mechanisms used by Al and theobromine to induce caffeine biosynthesis are similar. The cell suspensions of *C. arabica* are therefore a powerful model, very accessible with remarkable potential to study the mechanisms that regulate the synthesis of specific molecules. This leads to the modification of the metabolic pathways involved in the final production of secondary metabolites.

As an alternative, the use of microbial engineered organisms for the biosynthesis of plant natural products and derivatives has also been applied for the improvement of caffeine production. To find a better heterologous expression system for plant enzymes, *Saccharomyces cerevisiae* has been selected as the host due to a closer distance between plants and fungi [120]. Also, methylxanthines have been produced by feeding a methylated substrate (theophylline) to *Escherichia coli* cells expressing a bacterial demethylase [121]. This approach requires feeding high levels of methylated substrates to the engineered cells and may face limitations on the type and quantity of substrates that can be fed to or accessed by the host. 

In the first report of caffeine production in microbial systems, Jin and colleagues described engineered yeast strains that were able to synthesize caffeine (380 μg/L) from xanthosine fed to the cultures [122]. The authors co-expressed the native *N*-methyltransferases from *C. arabica* (CaXMT1) and caffeine synthase (TCS1) in yeast and fed as a substrate xanthosine for the production of caffeine. When they used the endogenous yeast xanthosine, caffeine was not detected and only found when exogenous xanthosine was used. This might be because the endogenous xanthosine is mainly used in primary metabolism and suggests that the endogenous level of xanthosine in yeast is insufficient to support the heterologous biosynthesis of caffeine. The caffeine metabolic pathway was completed by expressing genes from *C. arabica* and by identifying enzyme variants exhibiting the desired activity in the microbial host chosen for caffeine production [123]. In this study, *S. cerevisiae* was used as a system to study the co-expression of different *N*-methyltransferases. The authors were able to demonstrate a re-direction of the flux to an alternative pathway and to develop strains that supported the production of plant purine alkaloids and different methylxanthines. Upon re-directing the metabolic flux from the central metabolism into the target caffeine pathway, it was possible to specifically convert the endogenous xanthosine into 7-methylxanthosine and then to 7-methylxanthine. More specifically, via the redirection of xanthosine metabolic flux into the caffeine pathway, the de novo synthesis of caffeine in yeast hosts was attained (8.5 μg/L after 3 days) [123]. 

Different microbial platforms can also be used for the characterization of novel enzymes for the bioremediation of xanthine-derived compounds, such as degrading caffeine in wastewater [124]. These studies are valuable examples to establish the potential of microbial biosynthesis as a modern and flexible technology to address challenges in producing valuable plant-derived compounds.

Despite the progress of the recent years in the development of alternative systems to produce and study caffeine pathway, many important questions and challenges remain in this field. The use of genomic tools to improve the production of secondary metabolites may provide answers that will allow the transformation of crops, such as *Coffea*, to increase the production of secondary metabolites. 

## 5. Plant Glycosyltransferases: Versatile Players in Biotechnology

**Figure 2:**
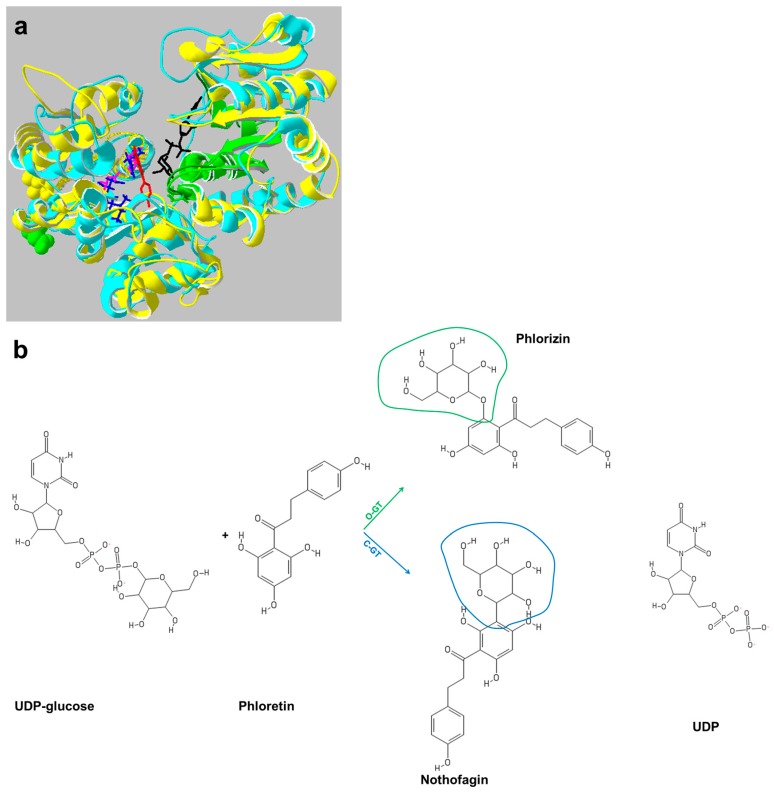
X-ray crystal structure of O-GT from *Vitis vinifera* (VvO-GT, PDB: 2C1Z) superimposed on the homology model of C-GT from *Oryza sativa* (**a**) and the GT-catalyzed reaction (**b**). (**a**) Yellow/light green, VvO-GT; left, N-terminal domain showing catalytic H20 and D119 residues (pink); right, C-terminal domain showing the green PSPG motif that binds the donor sugar (black). Here the donor analogue is UDP-2-deoxy-2-fluoro-d-glucose; Red, acceptor (kaempferol). Turquoise/dark green, rice-CGT; left, N-terminal domain showing H24, D120 and I121 residues (blue); right, C-terminal domain showing the dark green PSPG motif that binds donor sugar (black). Please note that the two imidazole rings of H20 and H24 are at almost right-angles to each other. The homology model was created by I-TASSER [150] and structures visualized by Swiss PDB Deepview 4.1 [151]. (**b**) Reactions catalyzed by O-GT and C-GT. The nucleotide sugar donor (UDP-glucose) reacts with phloretin to give the respective *O*- or *C*-glycosides.

Sugar conjugation (i.e., glycosylation) is one of the modifications adding diversity to the rich palette of plant secondary metabolites, thereby increasing their stability, as well as solubility and bioavailability [125]. Besides these effects on the chemical properties of plant secondary metabolites, glycosylation also determines compartmentation: an emblematic example is given by monolignols and anthocyanins, whose glycosylated forms are stored in the vacuole [126,127]. Glycosylation of plant secondary metabolites is catalyzed by a group of enzymes known as GTs that belong to family 1 of the Carbohydrate-Active enZYme database (CAZY) [128] and are referred to as UDP (uridine diphosphate) GTs, or simply as UGTs. Figure 2 shows the crystal structure of a typical O-GT (catalyzing *O*-glycosylation) from *Vitis vinifera* (yellow/green), superimposed on a homology model of C-GT (catalyzing *C*-glycosylation, see below) from *Oryza sativa* (turquoise). Since they both use UDP sugars as donors, they belong to the Leloir-type GTs. Among nucleotide sugars, UDP-glucose is the most common donor; however, other activated sugars can be used by UGTs, notably UDP-galactose, UDP-rhamnose, UDP-xylose and UDP-glucuronic acid [129]. The UGTs have a GT-B fold and an inverting mechanism (they are placed within clade II), meaning that the anomeric configuration of the product is inverted with respect to the nucleotide sugar donor [130]. Glycosyltrasferases partaking in the glycosylation of plant secondary metabolites display a conserved motif of ca. 40 amino acids towards the C-terminus, called the PSPG (plant secondary product glycosyltransferases) box (Figure 2, green) [131]. The UGTs containing the PSPG box are soluble enzymes [132], a feature that is very useful for expression in heterologous hosts. The PSPG box recognizes the nucleotide sugar donor [133], while the N-terminus recognizes the secondary metabolite as a substrate (acceptor) (Figure 2, left domain). Conserved amino acids in the PSPG box of UGTs are important for the recognition of the acceptors; however, the less conserved residues are important for the catalytic activity: by exchanging the PSPG box of a *Catharanthus roseus* curcumin UGT with that of a tobacco UGT, the catalytic activity in the chimera was lost and it was restored only when a non-conserved arginine residue was mutated to cysteine (the original amino acid present in the curcumin UGT) [134]. 

As previously mentioned, plant UGTs possess a GT-B fold characterized by two adjacent β/α/β Rossmann-like domains facing each other and joined by a flexible linker [130]. This architecture establishes the presence of two well-separated domains mediating the recognition of the substrate and sugar donors; these domains can be swapped between UGTs to create active chimeras. Enzyme engineering strategies based on domain swapping have been used to create chimeric UGTs displaying improved catalytic activities and broader, more promiscuous substrate specificities. For example, chimeric UGTs obtained by exchanging domains between the *Arabidopsis* flavonol 3-*O*-glucosyltransferase AtUGT78D2 and flavonol 3-*O*-arabinosyltransferase AtUGT78D3 led to two chimeras showing both UDP-glucose and UDP-arabinose specificity [135]. The domain swapping strategies have however unveiled that the recognition of the acceptor substrate is often mediated by the contribution from both the N- and C-terminal regions of UGTs, as shown by the often unpredictable catalytic activities of chimeras towards the acceptors [136]. 

Approaches based on site-directed mutagenesis have also been applied to plant UGTs to identify the key amino acids responsible for the nucleotide sugar and substrate recognition. Site-directed mutagenesis of a UDP-dependent glucosyltransferase from red daisy, BpUGT94B1, showed the crucial role of an arginine residue outside the PSPG box in recognizing UDP-glucuronic acid and the importance of asparagine and aspartic acid residues in the substrate binding pocket, where they form a stabilizing H bond with the 4′-OH of the acceptor’s B ring [137]. Small differences in amino acids can influence the type of glycosylated secondary metabolites accumulated by plants. For example, recently, comparisons of UGT89A2, an enzyme responsible for the accumulation of dihydroxybenzoic acid glycosides in various *Arabidopsis* natural accessions, revealed that the amino acid at position 153 was correlated with the different metabolic phenotypes and the preferential selectivity towards UDP-xylose, or both UDP-glucose and UDP-xylose donors [138]. 

Protein engineering strategies involving site-directed mutagenesis is limited by the availability of X-ray structures. In the absence of structural information, chemical modification (CM) can provide an alternative or complementary technique to genetic modifications (GM) for the identification of critical binding and catalytic residues in the absence and presence of competitive inhibitors or substrates. In addition, CM can also be used for the enhancement in the catalytic properties (such as stability and activity) of enzymes. In contrast to GM that generally relies on only 20 amino acids, CM can, potentially, employ a limitless variety of modifiers for attachment to enzymes ([139] and references therein). In spite of certain advantages of CM vs. GM strategies, very little work has been reported on the chemical modification of GTs. In one pioneering structure-function study on limonoid GT (that reduces bitterness in citrus), the role of various residues was investigated by a variety of chemical modification strategies at various pHs, in the absence and presence of limonin. The study revealed that histidine and acidic residues were essential for the transfer of the d-glucopyranosyl unit and binding respectively, whereas a tryptophan residue had a role in maintaining the proper conformation of the active-site. On the other hand, cysteine and serine had no role in the catalytic functioning of the enzyme [140]. To the best of our knowledge, no studies have been reported on the enhancement in the activity and stability of GTs using chemical modification or immobilization on magnetic nanoparticles (MNP). Immobilization on MNP was shown to enhance protein stability, catalytic activity and volumetric productivity, with concomitant recycling of expensive enzymes to decrease the cost of the process on a commercial scale [141]. 

In this paragraph, we also discuss the C-GTs, a special type of Leloir enzymes catalyzing *C*-glycosylation (Figure 2, turquoise), a reaction involving the formation of C-C bonds between the acceptor and the sugar donor, thus resulting in more stable *C*-glycosides as compared to highly hydrolysis-sensitive *O*-glycosides [142]. C-GTs have attracted much attention since the resulting modified secondary metabolites display other interesting features beyond enhanced stability, notably increased antioxidant and anti-inflammatory activities, prevention of obesity and diabetes [143,144]. Bifunctional UGTs displaying *C*- and *O*-glucosyltransferase activities have also been identified in plants: in maize, a bifunctional UGT, UGT708A6, was shown to catalyze the formation of *C*-glycosides from 2-hydroxyflavanones and *O*-glycosides using flavanones [143]. Such bifunctional enzymes are valuable for further biochemical studies to understand the mechanism involved in the creation of more stable *C*-glycosidic bonds that are less susceptible to hydrolysis as compared to *O*-glycosidic bonds [144]. 

The homology models of rice C-GT (Figure 2, turquoise) and pear O-GT are built on the crystal structure of grape vine VvO-GT (Figure 2, yellow/green) in the presence of both the sugar donor and acceptor substrate. The structures show the involvement of histidine H15 and H24 (H20 in Vv) as catalytic base that deprotonates the substrate as in pear O-GT and rice C-GT, respectively. In addition, aspartic acid D118 and D120 (D119 in Vv) acts to enhance the p*K*a, as well as to keep the histidine in the proper orientation by stabilizing the protonated residue. The models further suggest that a shift from *O*- to *C*-glycoside activity is probably due to the disruption in aspartic acid to histidine configuration caused by isoleucine (Ile, I)-aspartic acid (Asp, D) and aspartic acid-isoleucine residues exchange between rice O-GT and pear C-GT [145].

Swapping of active site motifs (Ile-Asp and Asp-Ile) between a rice C-GT and a pear O-GT showed that I117D and D118I substitutions in the pear O-GT double mutant led to the 100% formation of the *C*-glucoside nothofagin. Although the double mutant (D120I/I121D) of pear O-GT containing rice C-GT motif formed less than 10% of *O*-glycoside, the I121D single mutant gave equal amounts of *O*- and *C*-glycosides. In both mutants there was a significant loss in specific activity, implying the involvement of other critical residues [145]. 

With the ever-increasing number of UGT crystal structures available, protein engineering strategies, such as amino acid substitution of bulky residues with smaller ones in the substrate binding pocket and molecular dynamics simulations will be powerful tools for the rational design of improved UGTs displaying broader substrate and donor specificities. In 2009 a rational design approach based on domain swapping was successfully applied to closely- and distantly-related UGTs, which led to 20 chimeras showing improved efficiencies and novel substrate specificities [136]. 

Such engineered enzymes can be produced in planta to create more stable and bioavailable secondary metabolites with potential interest and application in the pharmaceutical/nutraceutical industries. The rapid advances in the field of genome editing (for example using the CRISPR/Cas9 system [146]) enables exceedingly precise site-directed mutagenesis. Hence, the editing of plant UGTs represents a highly exciting and captivating area for the engineering of plant secondary metabolites to achieve enhanced stability and bioavailability. Glycoengineering of bioactive molecules by modified plant UGTs is more attractive than organic synthesis, due to numerous drawbacks that include unwanted anomers produced during the different steps, use of heavy metal catalysts and low yields [147].

Protein engineering strategies to produce novel GTs catalyzing the formation of more stable glycosidic bonds can be limited by, e.g., the still restricted number of plant C-GTs characterized. The technique of directed evolution (DE) may be more useful in such cases. To restrict the need to screen a large mutational library, variation of this semi-rational method can be employed [148]. For example, iterative site-saturation mutagenesis (ISM), where a critical amino acid is replaced by all other 19 amino acids at that position, can be used to select for the most efficient GT which can be further improved in the second round, by targeting another residue in the active-site. The process is continued iteratively till the enzyme with the desired properties is obtained. Surprisingly, ISM has been applied on only a handful of GTs, including a cyclodextrin GT, to identify critical residues involved in higher activity and improved substrate specificity [148,149] and references therein. 

## 6. How to Boost Secondary Metabolite Production in Plant Cell Cultures?

**Figure 3:**
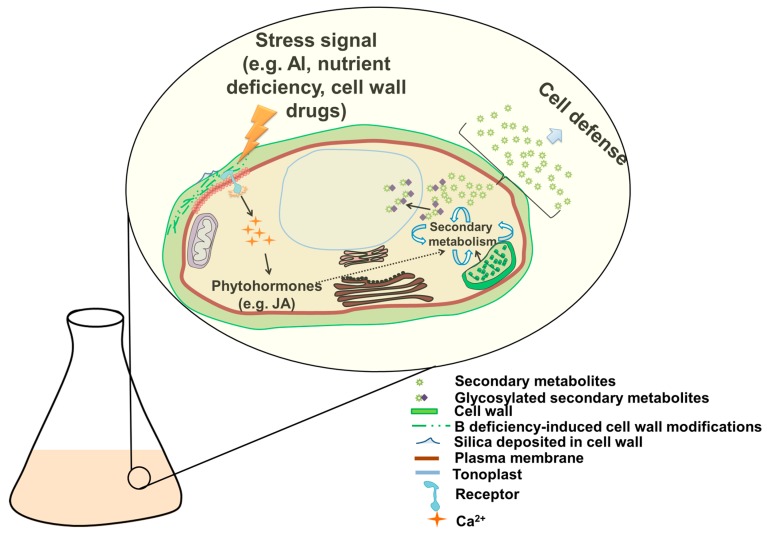
Cartoon depicting the effects of exogenous stresses on the cell wall and the subsequent production of secondary metabolites in plant cell cultures. Cell wall drugs, as well as micronutrient deficiency, silicon addition and/or Al toxicity affect the plant cell wall by inducing modifications. These changes are sensed by specific receptors at the interface between plasma membrane and cell wall which unleash a signaling cascade resulting in calcium accumulation and induction of specific phytohormones (e.g., jasmonic acid). These induce the production of secondary metabolites as a response to the stress. Precursors for specific secondary metabolite biosynthesis are provided by the chloroplast; glycosylated secondary metabolites are stored in the vacuole. The dotted arrow indicates an effect mediated by different players (transcription factors are an example).

Many of the molecules originating from secondary metabolism and described above are, regrettably, often present in small amounts within the cells. While this is acceptable from a biological point of view, biotechnology is expected to increase the production of these molecules. Enhancing the expression of genes associated with the synthesis of these metabolites is an obvious way forward, but not always possible due to the limited amount of information available. Alternatively, the production of specific secondary metabolites can be boosted by treating plants with environmental micro-stresses (Figure 3), as previously discussed for Al stress. Several evidences in the literature have demonstrated the existence of a cross-talk between the plant cell wall and the external environment. The cell wall integrity (CWI) signaling monitors the cell wall status in conditions of e.g., biotic stress and sends both chemical and physical signals to receptors located in the underlying plasma membrane [152,153]. Alterations at the cell wall-level are translated into signals through turgor pressure sensors (like mechanosensitive channels; Figure 3), which in their turn transform the physical signal into a chemical one (e.g., calcium influx). In case of abiotic stresses, the cell wall reacts in two main ways, either by increased plasticity (via the upregulation of expansins, xyloglucan endotransglucosylase/hydrolase, genes responsible for the synthesis of rhamnogalacturonan I in the pectin “hairy” regions), or by stiffening it (enhanced expression of lignin biosynthesis-related genes, i.e., candidates acting in the synthesis and polymerization of monolignols) [154]. 

The cross-talk existing between environment-cell wall is a feature found in other organisms synthesizing a cell wall, like yeast (where the CWI signaling was first described [155]) and fungi. Notably, in the latter, it was recently demonstrated that *Aspergillus nidulans* cell wall mutants (affected in the synthesis of mixed-linkage glucans) showed an increased expression of genes involved in the production of secondary metabolites and an enhanced production of sterigmatocystin [156]. These elements, i.e., the relationship cell wall-environment and cell wall integrity-secondary metabolites, lead to the conclusion that alterations affecting plant cell walls must have consequences on the production of secondary metabolites, either directly or indirectly. In support of this conclusion, the literature has shown that thale cress mutants impaired in secondary cell wall biosynthesis (i.e., mutations in secondary cell wall cellulose synthases *CesA8* and *CesA4*) display an increased production of secondary metabolites (camalexins and glucosinolates acting as antimicrobials) which protect against pathogen attack [157]. 

*Arabidopsis* mutants affected in primary cell wall biosynthesis (i.e., in the primary cellulose synthase *CesA3*) show also an increased resistance to pathogens mediated by the ethylene and jasmonic acid signaling pathway [158]. 

Alterations in the cell wall composition can be triggered via the use of cell wall drugs, for example the well-known cellulose biosynthesis inhibitors dichlobenil (DCB) and isoxaben (IXB) [159,160]. Convincing data in the literature have shown that it is possible to habituate plant cell cultures to grow in the presence of cell wall inhibitors by adopting a procedure consisting of transferring the cells to higher concentrations of the inhibitor in a step-wise manner [161,162]. Therefore, it would be interesting to obtain calli and cell suspension cultures of selected plants producing secondary metabolites of interest and quantify the production under conditions of habituation to a cell wall inhibitor. It is noteworthy in this respect that maize cells habituated to grow in the presence of DCB showed differences in cell wall composition and structure due to an increased phenolic metabolism (resulting in feruloyl-arabinoxylans cross-links strengthening the cellulose-deficient cell wall) [163]. The activation of the phenolic metabolism in habituated cells is interesting in case of targeted production of phenolic compounds, such as lignans. Plant biotechnologists may consider growing habituated cells of a specific (medicinal) plant and feed lignan biosynthetic precursors, such as those used in cell cultures of *Phyllanthus niruri*, i.e., ferulic acid or caffeic acid [164]. The combined action of habituation to cellulose biosynthesis inhibitors and the feeding of precursors may lead to an increased production of lignans, as compared to normal cells.

In this paragraph we will also discuss the role of the micronutrient boron (B) and the quasi-essential element Si, as they affect the properties of plant cell walls. B is involved in cross-linking the side chains of rhamnogalacturonan II in pectins and deficiencies in this micronutrient can therefore cause alterations in the cell wall structure, usually resulting in thickening and swelling due to an increase in the cell wall pore sizes [165]. In the literature, some papers have reported an alteration in plant secondary metabolites upon micronutrient deficiencies. For example, B deficiency in tobacco leaves induced the accumulation of soluble sugars, as well as of phenolic compounds (chlorogenic acids and caffeoyl polyamine conjugates) [166]; in olive trees grown in controlled chambers and in the field, B deficiency induced an increase in a distinct phenolic compound resembling caffeic acid (and referred to as compound A330) [167]. In the light of the known role that B has in the cell wall architecture, it would be interesting to establish whether the altered accumulation observed in these studies is due to cell wall modifications analogous to those described above for habituated cells. For example, one could expect that alterations in pectin structure induce compensatory mechanisms of wall strengthening which may activate secondary metabolic branches. In biotechnology, it could be possible to prepare cultures of plant cells growing under limiting B conditions; however, optimizations are necessary, as the micronutrient limitation will have an effect on the biomass yield.

Si, which is taken up by plants under the form of silicic acid Si(OH)_4_, has been reported as a booster of plant vigor and as a priming agent against several forms of stresses [168,169]. Si is deposited in the cell wall as biogenic opaline silica (SiO_2_) and this feature is important for the mechanical protection of plant tissues against e.g., the invasion of pathogens. However, besides this passive effect, Si was also shown to exert an active role, by preparing the response of plants to exogenous constraints. Several reports in the literature have demonstrated the latent role of Si, which becomes evident when an exogenous stress is applied. The plant defense arsenal is fully deployed at the onset of a stress but, before, the effects of Si on plant metabolism are not evident. There are a few exceptions, like rice, where the upregulation of 35 and downregulation of 121 transcription factors were observed under the presence of Si ([170] and references therein). 

A study on the medicinal plant *A. annua* L. showed that application of 400 kg ha^−1^ of silicate increased the trichome sizes and, consequently, the production of artemisinin [171]. Therefore, the addition of Si may be useful for the increased production of secondary metabolites produced in glandular trichomes.

Si has also shown many positive effects in plant tissue culture: calli formation is promoted, as well as somatic embryogenesis and shoot multiplication, while hyperhydricity is reduced [172]. 

In cell cultures of rice, Si improved the structural stability of the cell walls during the phases of expansion and divisions [173]. It will therefore be interesting to include this quasi-essential metalloid in the growth media of plant cell suspension cultures to enhance the production of secondary metabolites. The cultures will indeed show (1) increased vigor (senescence- delaying effect triggered by Si), (2) higher division rate (increased stabilization by association of Si with the cell wall), (3) primed secondary metabolism. Cell cultures of monocotyledonous plants (*Poaceae* in particular) will especially benefit from the addition of Si in the growth medium, since these representatives are known Si-accumulators [174]. 

## Figures and Tables

**Table 1:** Major chemical components of different *Artemisia* species.

Species	Chemical Constituents (%)	References
*A*. *annua*	Camphor (44), germacrene D (16), trans-pinocarveol (11), β-selinene (9), β-caryophyllene (9), artemisia ketone (3)	[18]
	Artemisia ketone (30.7), camphor (15.8)	[67]
	Artemisia ketone (35.7), α-pinene (16.5), 1,8-cineole (5.5)	[68]
	α-Pinene (7.33), camphene (5.68), sabinene (4.78), β-myrcene (22.41), 1,8-cineole (17.17), camphor (20.41)	[69]
	Camphor (17.74), α-pinene (9.66), germacrene D (7.55), 1,8-cineole (7.24), β-caryophyllene (7.02), artemisia ketone (6.26)	[70]
*A*. *sieberi*	Camphor (29.5), *cis*-thujone (22.58), 1,8-cineole (12.91), *trans*-thujone (10.60), camphene (5.05)	[71]
	Camphor (22.0), 1,8-cineole (19.3), *cis*-davanone (15.0), camphene (4.6), terpinene-4-ol (3.2)	[72]
	Spathulenol (30.42)	[73]
*A*. *monosperma*	β -Pinene (50.3), α-terpinolene (10.0), limonone (5.4), α-pinene (4.6)	[6]
	Butanoic acid (17.87)	[73]
*A*. *herba-alba*	Camphor (39.1), chrysanthenone (15.0), *cis*-thujone (7.8)	[6]
	Camphor (17–33), α-thujone (7–28), chrysanthenone (4–19)	[74]
	*Cis*-chrysanthenol (13.83), 1, 8-cineole (12.84), *cis*-limonene (12.57), α-terpinenol (6.97), γ-muurolene (4.50)	[75]
	α-Thujone (trace-47.1), camphor (5.6–30.0), chrysanthenone (trace-13.5), β -thujone (trace-9.2), 1,8-cineole (4.1–11.4)	[76]
	1,8-cineole (20.1), α-thujone (25.1), β -thujone (22.9), camphor (10.5)	[77]
	β -thujone (41.9), α-thujone (18.4), camphor (13.2)	[78]
	α-thujone (37.9), germacrene D (16.5), 1,8-cineole (8.4), β-thujone (7.8)	[79]
